# Development of adaptive pneumatic tourniquet systems based on minimal inflation pressure for upper limb surgeries

**DOI:** 10.1186/1475-925X-12-92

**Published:** 2013-09-23

**Authors:** Hong-yun Liu, Jun-yan Guo, Zheng-bo Zhang, Kai-yuan Li, Wei-dong Wang

**Affiliations:** 1 Department of Biomedical Engineering, Chinese PLA General Hospital, Fuxing Road, BeiJing, China; 2 Nursing Department, Chinese PLA General Hospital, Fuxing Road, BeiJing, China

**Keywords:** Pneumatic tourniquet, Upper limb, Arterial occlusion pressure, Microprocessor

## Abstract

**Background:**

Pneumatic tourniquets are medical devices that occlude blood flow to distal part of extremities and are commonly used in upper limb surgeries to provide a dry, clean and bloodless field. To decrease pressure-related injuries and potential risk of complications subjected to the high inflation pressure of pneumatic tourniquet, minimal inflation pressures are recommended.

**Methods:**

A new occlusion pressure mathematical model for the upper limb was established based on the correlation analysis between several possible influencing parameters and the minimal pneumatic tourniquet pressure at which the peripheral pulse disappeared was recorded using a digital plethysmograph. A prototype of an adaptive pneumatic tourniquet which automatically varies the pressure in the tourniquet cuff according to the above prediction model was developed for the upper limb which used the lowest possible inflation pressure to achieve occlusion. The prototype comprises a blood pressure monitoring module, an inflatable tourniquet cuff, and a pressure relief mechanism to maintain an optimal cuff inflation pressure. Simulation experiments were conducted to verify the function and stability of the designed adaptive pneumatic tourniquet and clinical experiments using volunteers were undertaken to evaluate the performance of the prototype design in achieving adequate haemostasis in the upper limb.

**Results:**

Results demonstrated that the mean arterial occlusion pressure was 152.3 ± 16.7 mmHg, obviously below the 250 to 300 mmHg previously recommended (J Bone Joint Surg Br 68:625-628, 1986 and Arthroscopy 11:307–311, 1995).

**Conclusions:**

In conclusion, this adaptive method and apparatus which can provide minimal inflation pressure may be a clinically practical alternative for upper limb surgery performed with pneumatic tourniquets.

## Background

In modern war conditions, extremity trauma with arterial blood loss is common [1]. Tourniquets have been identified as the most reasonable option for controlling extremity hemorrhage. Apart from that, pneumatic tourniquets are frequently used in field of extremity surgeries, venous puncture, regional anesthesia, and other situations of life or limb threatening [2]. The application of pneumatic tourniquets decreases the incidence of technical difficulties, but it is not without its complications [3,4]. Injuries resulting from pneumatic tourniquet use are commonly pressure related, and can also be caused by excessive tourniquet time, use of the lowest possible pressure is recommended [3-6].

The pressure to which a pneumatic tourniquet cuff should be inflated depends on a number of variables, including the patient’s age, skin, blood pressure and the shape and size of the extremity in question, as well as the dimensions of the cuff. However, many surgeons still use a standard pressure based on experiences, or they choose a cuff pressure using systolic blood pressure plus a standard margin or multiple in consideration of changing conditions during surgery [7,8]. As is known to all, blood pressure is not always constant and can vary with conditions, and pneumatic tourniquets based on these methods could not respond to blood pressure changes; any necessary adjustments had to be made manually. Yoshinori Ishii reported a new pneumatic tourniquet, in which the inflation pressure is only determined by systolic blood pressure detected by an additional vital information monitor, and this made the system more complicated [9,10]. Tourniquet systems developed by McEwen JA et al. [11] using limb occlusion pressure (LOP) to stop the arterial blood flow, which detected by an added blood flow transducer. Bahattin Tuncali also presented an estimation of tourniquet cuff inflation pressure based on systolic blood pressure and tissue padding coefficients, but was not applied comprehensively due to its invasive method [6,12]. Arterial occlusion pressure (AOP) is the pressure at which arterial blood flow is occluded with a specific pneumatic tourniquet cuff at a specific time in a specific limb [2]. Tourniquet inflation pressure (TIP), which is the pressure of cuff inflated has been shown to be useful in optimizing pneumatic tourniquets [12]. Current literature suggests that the complicate process of TIP’s determination method however is time consuming, labour intensive and skill required and despite its proven technical benefit, has been increased workload of doctors and nurses in clinical practice [3,12]. Results of these kinds of estimation are suboptimal because of the variable correlation relationship between TIPs and several possible influencing parameters such as body mass index (BMI), extremity circumference, arterial diameter, blood flow velocity, blood pressures and so on.

The aim of this study was to establish a mathematic model that predicts the upper limb’s minimal effective pneumatic TIP and to use this model in a prototype adaptive pneumatic tourniquet. To be applied in a comparative study involving this new system and conventional tourniquet systems while used on patients scheduled for upper limb surgeries, the effectiveness of the new designed adaptive pneumatic tourniquet was evaluated and verified.

## Methods

### Establishment of a mathematical model for AOP

70 healthy soldiers were recruited with Ethics Committee of Chinese PLA General Hospital approval, and informed consent was obtained from these volunteers (Table 1). The body mass index (BMI) was obtained through measuring the body weight and height of 70 volunteers; upper extremity circumference was measured with a tape measure at the medial side of the biceps brachii, about 10 cm above the anterior elbow; arterial diameter, blood flow velocity and blood pressure of 70 healthy soldiers at supine position were measured and recorded with a colour Doppler ultrasonography blood flow imaging system (ALT5000, PHILIPS) and an electronic sphygmomanometer (HEM-7011, OMRON) respectively. Simultaneously, the AOP at which the distal upper limp circulation is occluded were studied in 70 volunteers undergoing experiment through a digital plethysmograph. The tourniquet cuff used in the experiment was contoured and standardized as 86 cm long and 11 cm wide.

**Table 1:** 70 healthy soldiers’ demographics

**Parameter**	**Value**
**Age**	23 ± 4
**Gender**	40 men, 30 women
**Weight**	(60 ± 12) kg
**Height**	(169 ± 5) cm
**BMI**	(21 ± 4) kg/m^2^

Data are expressed as mean ± SD.

Before the experiment commenced, the digital plethysmograph was applied to the index finger of left hand and the pneumatic tourniquet cuff was inflated slowly until the photoplethysmography disappeared on the oscilloscope. The corresponding AOP monitored by GE Druck DPI 610 portable pressure calibrator were recorded. Using statistical software SPSS13.0, the correlation between the above influencing parameters and AOP was analyzed and the Pearson correlation coefficients were obtained (Table 2). Eventually, the predicting model for AOP (mmHg) was established as following:

AOP=17.986+3.158X1+0.408X2

**Table 2:** Results of single factor analysis for arterial occlusion pressure of upper extremities

	$\bar{X}\pm \mathrm{SD}$	**Pearson correlation coefficients *r***	**P**
**Systolic blood pressure (mmHg)**	115 ± 13	0.657	0.00
**Diastolic blood pressure (mmHg)**	67 ± 8	0.424	0.00
**Upper extremity circumference (cm)**	23 ± 3	0.716	0.00
**Body mass index (kg/m**^**2**^**)**	21 ± 4	0.159	0.19
**Arterial diameter (mm)**	3 ± 0.5	0.436	0.00
**Blood flow velocity (cm/s)**	84 ± 18	0.280	0.02

Coefficients between AOP and systolic blood pressure, diastolic blood pressure, upper extremity circumference, BMI, arterial diameter and blood flow velocity were analyzed with Pearson correlation. The mean difference is significant at the 0.05 level.

Where X1 = upper extremity circumference (cm) and X2 = systolic blood pressure (SBP, mmHg).

### Development of the adaptive pneumatic tourniquet based on the new AOP model

**Figure 1:**
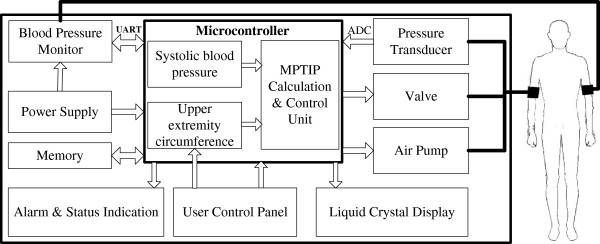
**Block diagram of adaptive pneumatic tourniquet based on AOP model.** The adaptive pneumatic tourniquet contains a blood pressure monitoring module, some peripheral circuits, an inflatable tourniquet cuff, a pressure regulator mechanism responsive to the blood pressure and upper limb circumference for adjusting TIP.

Based on the above established AOP model, a prototype adaptive pneumatic tourniquet system was developed in which the TIP was determined by upper extremity circumference and systolic blood pressure was developed. This prototype (as shown in Figure 1) contains a blood pressure monitoring module, an inflatable tourniquet cuff, and a pressure relief mechanism to maintain cuff pressure approximately at minimal pneumatic tourniquet inflation pressure (MPTIP) to stop the blood flow of upper limb artery and provide a bloodless surgical field. In other words, cuff pressure is adapted to the patient’s upper limb circumference and systolic blood pressure as the function described above.

**Figure 2:**
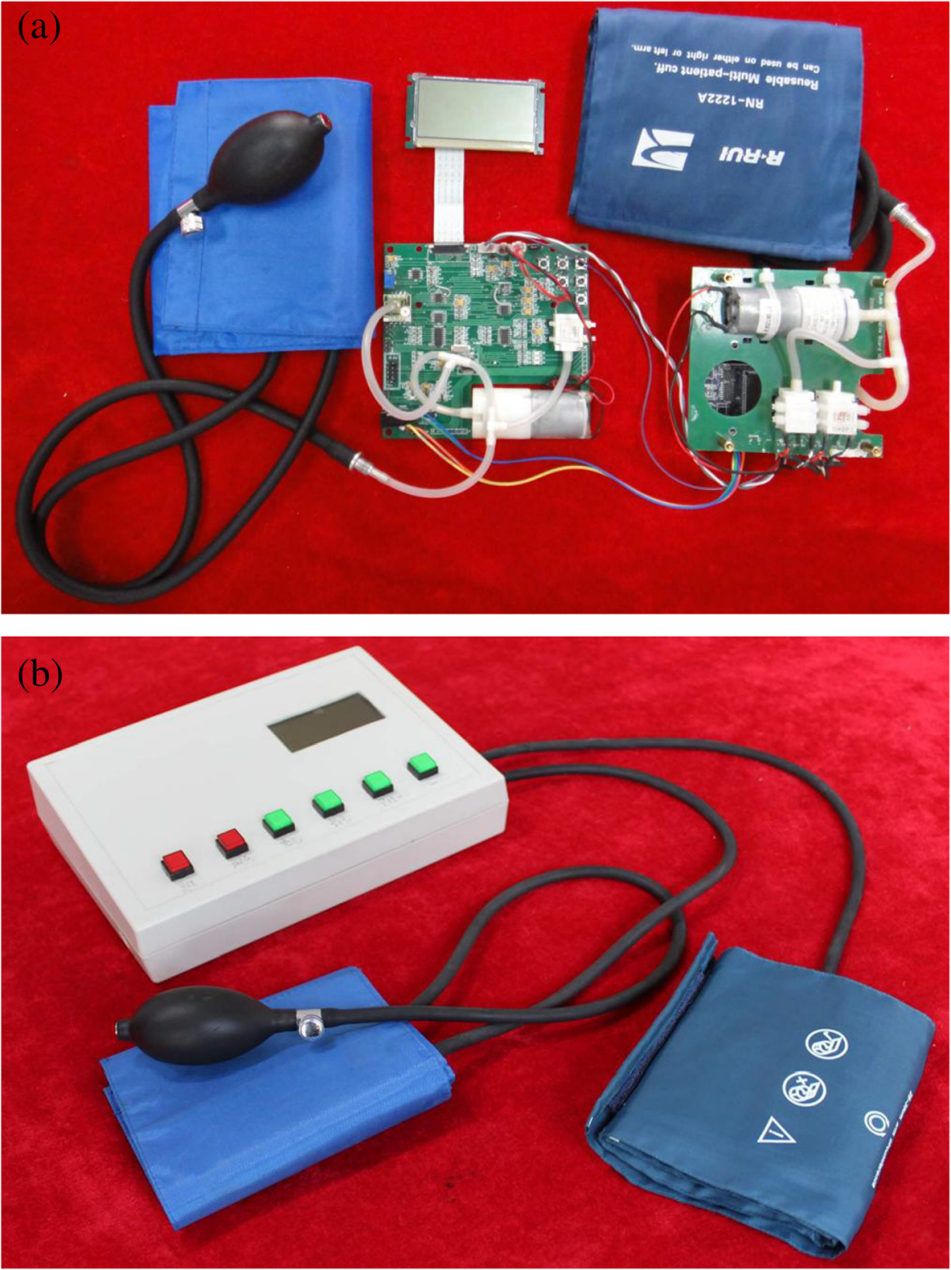
**Prototype of designed adaptive pneumatic tourniquet.** The new system consists of two circuit boards (blood pressure monitor board and core circuit board) **a)** architecture of internal circuits, **b)** physical view of prototype according to the present implementation.

The non-invasive blood pressure monitor module consists of three main parts: external hardware, analog circuit, and microcontroller. An oscillometry method is used to periodically estimate the patient’s mean arterial blood pressure through inflating and deflating blood pressure cuff placed on the patient’s arm alternatively, and employ an algorithm to extrapolate values of patient’s systolic blood pressure. Simultaneously, the upper extremity circumference of patient is measured manually with a tape measure at the medial side of the biceps brachii and entered as an input to the calculation algorithm through user control panel. The microcontroller obtains upper extremity circumference and the systolic blood pressure values through Universal Asynchronous Receiver/Transmitter (UART) communication interface and computes AOP with the above prediction model. A margin error of 10 mmHg is added on the AOP to obtain the MPTIP. Corresponding programs are preset in the MPTIP & control unit of the microcontroller. Pneumatic tourniquet cuff pressure is detected by the pressure transducer, the output analog signal from which is converted to digital form through analog to digital converter (ADC) suitable for interpretation by microcontroller. Microcontroller compares the calculated TIP and the detected TIP and control the air pump to inflate if the TIP falls below the calculated TIP, or produce a signal to trigger the valve for deflating is TIP exceeds the calculated TIP. The power supply module provides the system with about 3.0 V voltages and provides sustainable energy savings across the network by monitoring energy usage and directing low-power states and shutdown times. The parameters of blood pressure, tourniquet cuff pressure and upper extremity circumference are displayed on the liquid crystal display (LCD) and are electronically coupled to microcontroller which has an associated memory. Alarm and status indication module provide the surgeon with series of audible or visual alarms to warn them of hazardous situations such as faulty operation, tourniquet cuff pressure out of range, long lasting tourniquet time and so on. Eventually, the prototype of the new adaptive pneumatic tourniquet using minimal inflation pressure was integrated (Figure 2) based on the block diagram.

### Verification for the performance of blood pressure monitor

To verify the performance of the non-invasive blood pressure (NIBP) monitor module, a Regel BP-SiM NIBP simulator which incorporates a range of custom settings that include a variety of adult NIBP pressures was applied. 7 standard pressure points were selected to simulate the blood pressure with a constant heart rate at 80 beats per minute. The standard blood pressure and heart rate for each point generated through the simulator and each was sampled 10 times by the NIBP monitor. Data of blood pressure and heart rate was recorded and analyzed.

### Performance test of the developed adaptive pneumatic tourniquet prototype

Adult patients (N = 20) scheduled for upper limb surgery were recruited for the performance study. Clinical protocols used were cleared by the Ethics Committee of Chinese PLA General Hospital and consent forms were completed and signed by each patient. The 20 patients were randomly divided into two groups: MPTIP group (A1) and the conventional pneumatic tourniquet group (A2) (Table 3). The tourniquet was applied to the arm undergoing surgery while the other arm was used to monitor blood pressure. Anesthesia was induced and the surgery commenced with TIP recorded in all groups for analysis. In the procedure of surgery, the width of the tourniquet cuff was both 11 cm in all patients.

**Table 3:** 20 patients’ demographics

	**A1 (n = 10)**	**A2 (n = 10)**
**Age**	31 ± 13	35 ± 13
**Gender**	7 men, 3 women	5 men, 5 women
**Upper extremity circumference**	(25 ± 3) cm	(24 ± 3) cm
**Tourniquet time**	108 ± 16 min	113 ± 20 min

Data are expressed as mean ± SD.

In group A1, the new designed adaptive pneumatic tourniquet which includes a NIBP monitor was applied to occlude the arterial blood flow with possible minimal inflation pressure. The upper extremity circumference of patients was measured before the surgery started and tourniquet cuff pressure renewing interval was pre-set. In group A2, the 2500ELC tourniquet system (VBM, Germany) was used to inflate the tourniquet cuff with conventional pressures 250 mmHg that are routinely used for upper limb surgery in our hospital. The vital information monitor, PM8000 (Mindray, China) adopts a simple cuff to detect the blood pressure of the patients. The interval of the measurement of blood pressure was set at 2.5 minutes for both groups. Systolic blood pressures and TIP were recorded at 0, 10, 20, 30, 40, 50 and 60 minutes. The surgeon evaluated the quality of occlusion with the method used by Ishii et al. [3,8,9]. Examination of signs for any complications about all patients was conducted after the procedure of surgery. Data was analyzed by Mann–Whitney U test between two groups and a value of P < 0.05 was deemed as statistically significant.

## Results

### Predicted regression equation of AOP

From Table 2 we learned that the AOP and systolic blood pressure (*r* = 0.657, *p* < 0.01), diastolic blood pressure (*r* = 0.424, *p* < 0.01), upper extremity circumference (*r* = 0.716, *p* < 0.01), arterial diameter (*r* = 0.436, *p* < 0.01), blood flow velocity (*r* = 0.280, *p* < 0.05) are positively correlated with significance. Multiple stepwise regression analysis showed that only upper extremity circumference (X1) and systolic blood pressure (X2) enter the regression equation through taking the above positively correlated parameters as independent variables. The predicting model for AOP (mmHg) was obtained [*AOP* = 17.986 + 3.158*X*1 + 0.408*X*2,X1 denotes upper extremity circumference (cm) and X2 denotes SBP (mmHg)] (Table 3), and the mean AOP for 70 healthy volunteers is 139 ± 17 mmHg.

### Simulation and clinical experiments

Experimentally determined heart beat rate measured using the system blood pressure monitor was 80 ±2 bpm for a simulator setting of 80 bpm. The blood pressure values obtained for the seven standard simulator set-point values were slightly higher than the set-points (typically 4 mmHg above set-point value) indicating a small systematic error in either the simulator or the monitor values.

**Figure 3:**
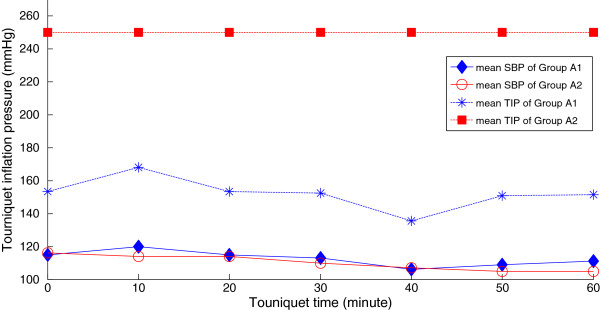
**SBP and corresponding applied TIP in group A1 and A2.** SBP and corresponding applied TIP in group A1 and A2. In group A1 new designed tourniquet system based on the AOP prediction model allowed not different SBP and significantly lower TIP in the procedure of surgery compared with group A2. SBP were 113 ± 5 mmHg in group A1 and 110 ± 5 mmHg in group A2; TIP were 152 ± 17 mmHg (range, 135-168 mmHg) in group A1 versus constantly 250 mmHg in group A2.

20 patients scheduled for upper limb surgeries participated in the experiment were no significant difference in demographic data (Table 4) between 2 groups. In the procedure of surgery, mean systolic blood pressures of patients were 113 ± 5 mmHg in group A1 and 110 ± 5 mmHg in group A2; for consideration of any changing situations during surgery, a margin of error of 10 mmHg was added on the predicted AOP and the average pneumatic TIP were 152 ± 17 mmHg (range, 135–168 mmHg) in group A1 versus constantly 250 mmHg in group A2. SBP were not different at 0, 10, 20, 30, 40, 50 and 60 minutes between 2 groups but with significant difference (*P* < 0.01) in pneumatic TIP at all stages of tourniquet time (Figure 3). Blood flow of artery was completely occluded in 20 cases in the procedure of surgery and surgical fields were all evaluated as satisfactory in both groups through questionnaire to surgeon who were blinded to the corresponding group. Most importantly, no complications including pain, and nerve, muscle, and skin injuries occurred during or after surgery for both group.

**Table 4:** Multiple stepwise regression analysis for AOP of upper extremities

**Model**	**Unstandardized coefficients**		**Standardized coefficients**	**t**	**Sig.**
	**B**	**Std. error**	**Beta**		
**(Constant)**	17.986	12.967		1.387	0.17
**X**_**1**_	3.158	0.680	0.5	4.647	0.000
**X**_**2**_	0.408	0.136	0.323	2.998	0.004

a → Dependent variable: AOP. Five parameters (systolic blood pressure, diastolic blood pressure, upper extremity circumference, arterial diameter and blood flow velocity) entered into stepwise regression procedure as independent variables and the upper extremity circumference (X_1_) and SBP (X_2_) were found as the best predictors, which were highly correlated with the dependent variable AOP.

## Discussion

Although almost one hundred years has been passed since Cushing introduced the pneumatic tourniquet, the safe limits for duration of tourniquet ischemia and optimal TIP are still being discussed [2-5,13,14]. The traditional recommended maximum safe tourniquet pressure for upper limb surgery is 250–300 mmHg for adult patients [7,15]. To determine the minimal inflation pressure for the upper limb some researchers indicated that a margin of error of 50–100 mmHg should be added on the SBP or twice the SBP to achieve bloodless field in consideration of changing conditions in the procedure of surgery [7,16-19]. The Association of Surgical Technologies recommended SBP plus 50 mmHg for upper limb tourniquet but Kam et al. suggested 50–150 mmHg above SBP [19]. Other investigators suggest that TIP should be set according to the AOP [20-22]. In Tuncali’s recent study [12], he developed an AOP equation [AOP = (SBP + 10)/K_TP_, where K_TP_ is a dimensionless quantity denotes the tissue padding coefficient] based on SBP and extremity circumferences in optimizing pneumatic TIP and Noordin et al. presented the view of using LOP without margin to occlude blood flow in their study [11,23]. Both AOP and limb occlusion pressure are the minimum pressure required in tourniquet cuff to stop the flow of arterial blood into the limb distal [12,23]. The current guidelines from Association of peri-Operative Registered Nurses (ARON) recommend that a safety margin of 40 mmHg be added for AOP below 130 mmHg, 60 mmHg be added for AOP between 131 mmHg and 190 mmHg, and 80 mmHg be added for AOP above 190 mmHg for adults [24].

Ideally, the pneumatic tourniquet should be applied at the lowest cuff inflation pressure and for the shortest duration time possible. Although the pneumatic tourniquet, minimal cuff inflation pressure of which based on the recommended values, synchronized SBP methods or AOP plus margin errors could achieve the goal of blood flow occlusion, the level of TIP that obtain hemostasis yet cannot avoid unnecessary pressure on soft tissue structures. The existing methods of determination AOP were time consuming, labour intensive, skill required or invasive therefore more complicated in clinical use. According to the previous study results, SBP, diastolic blood pressure, BMI, upper extremity circumference, artery diameter and blood flow velocity were selected as influencing parameters in our research, and an prediction regression AOP equation for upper limb was established based on the strong correlated influencing factors of SBP and upper extremity circumference. According to the equation, the prototype adaptive pneumatic tourniquet with possible minimal TIP was designed and implemented. Be different from the Ishii system and systems developed by McEwen JA, the prototype’s occlusion pressure was determined by both SBP and upper limb circumference of specific patient [8,9,22,24]. Performance test experiment showed that the prototype is able to monitor blood pressure periodically with tolerant errors and could stop the blood flow of upper limb effectively. In the controlled simulation experiment, using new developed AOP model and technique, significantly smaller TIP were obtained compared with those recommended values in previous literatures [7,15].

We all know that blood pressure of human varies with the changing of physical and mental state, environmental condition and other factors. In the adaptive pneumatic tourniquet’s current design version, blood pressure is monitored in a certain interval but not in real time. This may limit the possibilities for precisely and timely determination of AOP and corresponding minimal TIP. Because the designed prototype is not fully developed and perfected, only preliminary experiment on small sample volume was conducted. In the next step, a beat to beat blood monitor technique and electrical safety factors will be taken into consideration to optimize the developed prototype, with further clinical controlled experiments conducted, the more accurate upper limb AOP model and optimized minimal TIP will be established and obtained, and the adaptive pneumatic tourniquet for upper limb surgery integrated the above technologies will be relatively easy to be developed.

## Conclusions

In conclusion, a predicted regression model of upper limb AOP strong correlated SBP and upper limb circumference was established through investigating the relationship between AOP and BMI, upper extremity circumference, arterial diameter, blood flow velocity, and blood pressure. Based on this model, the prototype of adaptive pneumatic tourniquet system, the inflation pressure of which determined by the above AOP was developed for maintaining a bloodless surgical field in upper limb surgery. The new pneumatic tourniquet prototype was proved to be efficacy and safety in simulation experiment and surgeries with providing significantly smaller TIP than previously applied. By improving and perfecting, the method and apparatus which could reach real possible minimal inflation pressure may be clinically practical alternative for upper limb surgery performed with pneumatic tourniquets.

## Abbreviations

AOP: Arterial occlusion pressure; TIP: Tourniquet inflation pressure; BMI: Body mass index; MPTIP: Minimal pneumatic tourniquet inflation pressure; LOP: Limb occlusion pressure; UART: Universal Asynchronous receiver/transmitter; ADC: Analog to digital converter; LCD: Liquid crystal display; NIBP: Non-invasive blood pressure; SBP: Systolic blood pressure.

## Competing interests

The authors declare that they have no competing financial interests.

## Authors’ contributions

H-YL designed the prototype of the adaptive pneumatic tourniquet, conducted the simulation and clinical experiments and drafted the manuscript; J-YG carried on the simulation and clinical experiments and acquired experimental data; Z-BZ designed the basic analog circuits and gave a careful proofread to correct those grammar and usage errors; K-YL programming the prototype and analyzed and interpreted experimental data; W-DW have been involved in revising the manuscript critically for important intellectual content and have given final approval of the version to be published. All authors read and approved the final manuscript.
